# Coupling Plant-Derived Cyclotides to Metal Surfaces: An Antibacterial and Antibiofilm Study

**DOI:** 10.3390/ijms19030793

**Published:** 2018-03-09

**Authors:** Pan Cao, Ying Yang, Fidelia Ijeoma Uche, Sarah Ruth Hart, Wen-Wu Li, Chengqing Yuan

**Affiliations:** 1 School of Energy and Power Engineering, Wuhan University of Technology, Wuhan 430063, China; caop@whut.edu.cn; 2 Institute for Science and Technology in Medicine, Keele University, Stoke-on-Trent ST4 7QB, UK; y.yang@keele.ac.uk (Y.Y.); f.i.uche@keele.ac.uk (F.I.U.); s.r.hart@keele.ac.uk (S.R.H.)

**Keywords:** cyclotides, surface modification, antibiofilm, antibacterial, polydopamine

## Abstract

Modification of metal surfaces with antimicrobial peptides is a promising approach to reduce bacterial adhesion. Here, cyclic peptides or cycloids, possessing remarkable stability and antimicrobial activities, were extracted and purified from *Viola philippica* Cav., and identified using mass spectrometry. Cyclotides were subsequently utilized to modify stainless steel surfaces via polydopamine-mediated coupling. The resulting cyclotide-modified surfaces were characterized by Fourier transform infrared (FTIR) spectroscopy and contact angle analysis. The antibacterial capacity of these cyclotides against *Staphylococcus aureus* was assessed by Alamar blue assay. The antibiofilm capacity of the modified surfaces was assessed by crystal violet assay, and scanning electron microscopy (SEM). A composite of Kalata b1, Varv A, Viba 15 and Viba 17 (P1); Varv E (P2); and Viphi G (P3) were isolated and identified. FTIR analysis of the modified surfaces demonstrated that cyclotides bound to the surfaces and induced reduction of contact angles. Antimicrobial effects showed an order P3 > P1 and P2, with P3-treated surfaces demonstrating the strongest antibiofilm capacity. SEM confirmed reduced biofilm formation for P3-treated surfaces. This study provides novel evidence for cyclotides as a new class for development of antibacterial and antibiofilm agents.

## 1. Introduction

Biofouling is an important issue in diverse industries, including medical device and food manufacture, aquaculture, and shipbuilding [1,2]. Primary colonization is initiated by adhesion and settlement of bacteria; these colonies grow to create a biofilm matrix which acts as a precursor to promote subsequent fouling by macrofoulants [2]. Biofilm formation on medical implants, such as contact lenses, urinary tract and cardiovascular catheters, and orthopaedic prostheses causes infection, with huge healthcare costs [1]. Similarly, the loss to the shipping industry caused by bio-organism attachment to vessel surfaces exceeds tens of billions of dollars a year [3], and causes significant environmental damage [4]. Two major strategies have been developed to overcome the problem of biofouling: (i) prevention of biofoulant attachment; and (ii) biofoulant breakdown methods [1,5]. Preventative strategies are appealing in comparison to allowing biofilms to form; in particular, surface modification using biological molecules such as antimicrobial peptides and enzymes [6,7,8] may reduce biofouling by creating bactericidal, bacteria-resistant, or bacteria-repelling surfaces [5] following the first strategy. For example, peptide-modified steel surfaces have been prepared by sharing electrons between metal and peptide disulfide bonds, resulting in reduced bacterial surface colonization by *Pseudomonas aeruginosa* and *Staphylococcus aureus* [9]. Our group has previously expressed recombinant fusion proteins containing a stainless steel-binding domain, which were used to further functionalize metal surfaces [10]. Furthermore, we examined the influence of the bioorganic modifier on surface topography for a synthetic peptide-modified 304 stainless steel [11].

The inherent binding properties of peptides can be exploited to enable attachment to metal surfaces; however, coupling agents such as dopamine (DA) can enhance the binding between peptides/enzymes and metal surfaces [12,13,14]. Dopamine readily polymerizes on multiple solid surface types in a weak alkaline solution under oxygen [12]. Dopamine self-polymerization reactions to form polydopamine have been used to improve the efficiency of surface modification with antimicrobial lipopeptides [15], the antibacterial enzyme lysostaphin [16], and two synthetic 15-mer peptides for the prevention of biofilm formation [17]. Dopamine self-polymerization thus provides a high-efficiency, simple, and versatile means of derivatizing metal surfaces with a wide range of molecules.

**Figure 1:**
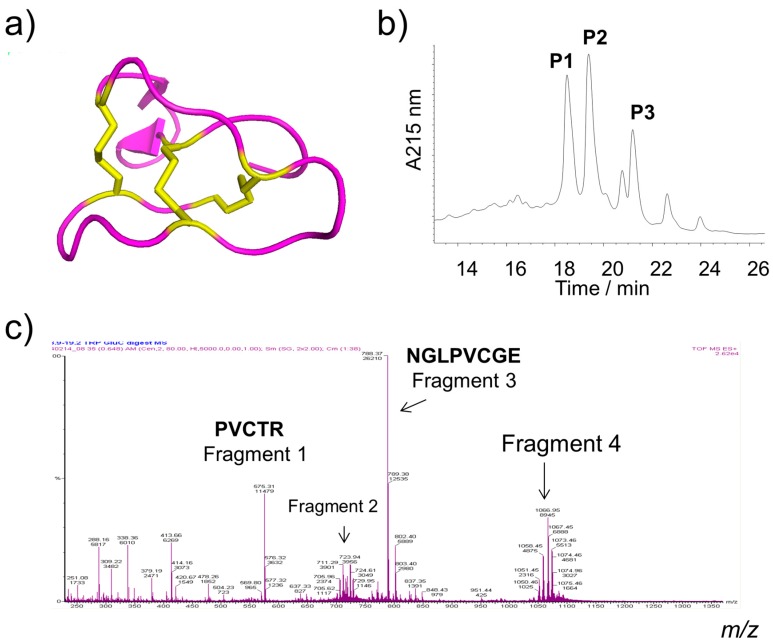

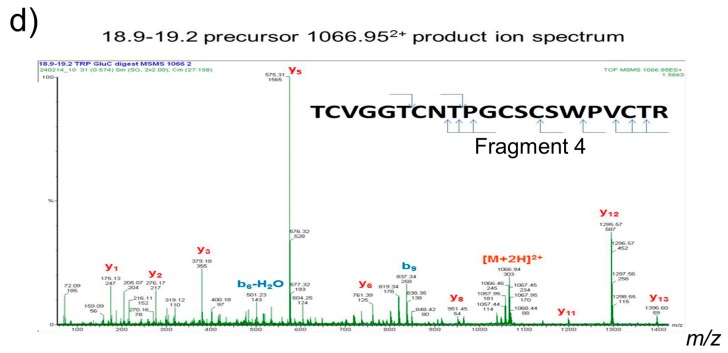
3D structure, purification and identification of cyclotides. (**a**) 3D cartoon structure of kalata b1 (PDB ID:1KAL) illustrating the knotted pattern with three internal disulfide bonds (yellow and stick); (**b**) Semi-preparative HPLC showing elution of cyclotides from *V. philippica*, P1, P2, and P3; (**c**) A survey mass spectrum of four digested peptide fragments of Varv A; (**d**) Product ion spectrum of fragment 4 (derived from Varv A).

A potential pitfall in the application of immobilized linear peptides or enzymes on metal surfaces is their susceptibility to the action of proteases and consequent degradation in biological environments. This suggests that antimicrobial peptides with increased stability are desirable targets for such applications. Cyclotides are macrocyclic peptides derived from plants such as Violaceae, Rubiaceae, and Cucurbitaceae families. They are typically ~30 mers, featuring three disulfide bonds and arranged in a knotted pattern (Figure 1a). This structural feature bestows the cyclotides with remarkable stability against proteolytic hydrolysis, as well as thermal and chemical denaturation [18,19]. Cyclotides exhibit a broad range of bioactivities including anticancer [20,21], anti-HIV, insecticidal, antibacterial [22,23], and anti-biofouling effects [24].

In this study, cyclotides were isolated and characterized from a Chinese medicinal plant *Viola philippica* Cav. and used to modify stainless steel surfaces with dopamine as a coupling agent. Multiple surface characterization assessments, as well as antibacterial and antibiofilm assays, were conducted to demonstrate modification of metal surfaces as well as antibacterial and antibiofilm ability. This study suggests new potential peptide sources for metal surface modification towards development of antibiofilm devices.

## 2. Results

### 2.1. Isolation and Identification of Cyclotides

Cyclotides were extracted from *V. philippica* and subjected to silica gel column chromatography. Cyclotide-enriched sub-fractions were further purified by semi-preparative reversed-phase high performance liquid chromatography (HPLC). Three cyclotides, appearing at 18, 19, and 21 min, were collected, lyophilized, and denoted as P1, P2, and P3, respectively (Figure 1b). The elution times of these peptides indicated that the order of hydrophobicity of the three cyclotides is as follows: P3 > P2 > P1.

The primary structures (sequence) of the cyclotides were identified by enzymatic digestion followed by liquid chromatography tandem mass spectrometry (LC-MS) and comparison of alignments to published cyclotide sequences. P1 was identified as being comprised of a mixture of Kalata b1 (Figure 1a), Varv A (Figure 1c,d), Viba 15, and Viba 17. P2 and P3 were identified as Varv E (Cycloviolacin O12), and Viphi G, respectively. All these cyclotides were characterized from this plant in agreement with a previous study [20]. The sequences, experimental masses, and net charges of these cyclotides are listed in Table 1. P3 (Viphi G) has a positive charge, while others are neutral. P3 has the greatest GRAVY (grand average of hydropathicity) value for its linear form, consistent with its hydrophobic behavior on HPLC (Figure 1b).

### 2.2. Characterization of Cyclotide-Modified Metal Surfaces

#### 2.2.1. Fourier Transform Infrared (FTIR) Spectroscopy

**Figure 2:**
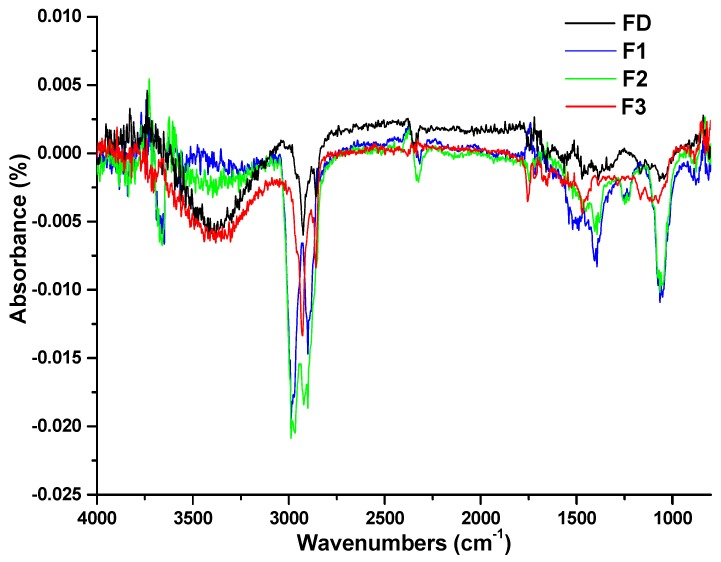
FTIR spectra of dopamine- and cyclotides-treated metal disk samples. For FD, spectra were collected using an untreated sample as background; for F1, F2, and F3, FTIR spectra were collected using FD as background. Metal disk samples treated with dopamine (DA), P1, P2, and P3 were denoted as FD, F1, F2, and F3, respectively.

FTIR spectroscopy is used here to identify functional groups on peptide-derivatized stainless steel. Metal samples treated with DA, P1, P2, and P3 were denoted as FD, F1, F2, and F3, respectively. In cyclotide-treated metal samples F1–F3, but not FD, distinct peaks appeared at 1690 and 1520 cm^−1^, sharper peaks in the region of 1300–1400 cm^−1^, and strong absorption around 1100 cm^−1^ (Figure 2). The presence of these peaks exclusively in cyclotide-treated surfaces supports cyclotides being bound to the metal surfaces.

#### 2.2.2. Contact Angle Analysis

**Figure 3:**
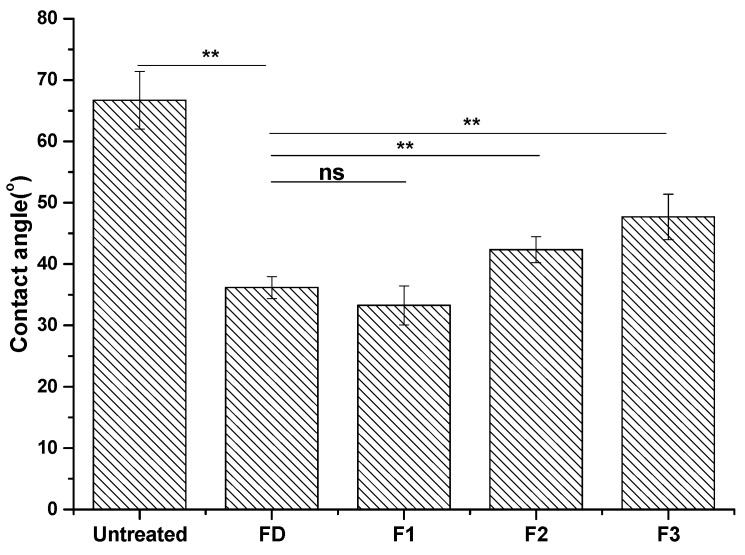
Contact angles of 304 stainless steel samples before and after surface treatment by DA and three cyclotides (P1, P2, and P3). Metal disk samples treated with DA, P1, P2, and P3 were denoted as FD, F1, F2, and F3, respectively. One-way analysis of variance (ANOVA) was used to compare groups (ns: not significant; ** *p* < 0.01, *n* = 4). Error bars indicate the standard deviation of the four independent measurements.

The contact angles of DA- and cyclotide-treated samples decreased compared with that of the untreated samples (Figure 3). F3 showed the highest hydrophobicity, followed by F2. F1 possessed a similar wettability to that of dopamine-treated samples (FD), which can be ascribed to the noted difference in the hydrophobicity of cyclotides (Table 1, Figure 1b).

### 2.3. Antibacterial and Antibiofilm Activity

**Figure 4:**
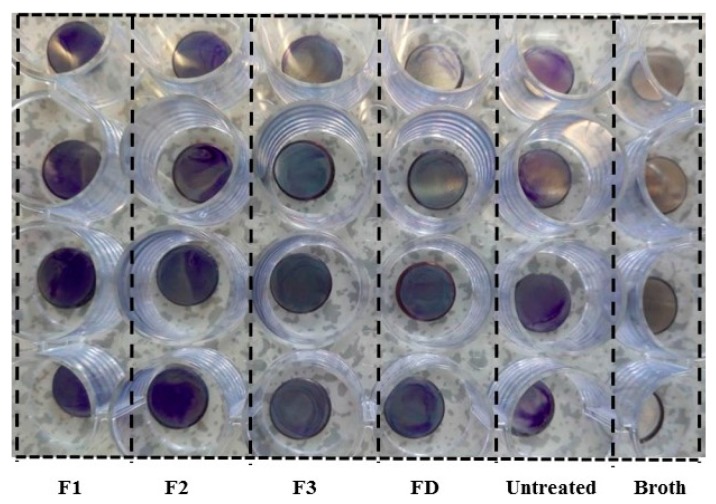
Replicate images (*n =* 4) of cyclotide- and DA-modified metal surfaces (F1, F2, F3, and FD) after incubation with *S. aureus*. Metal disk samples treated with DA, P1, P2, and P3 were denoted as FD, F1, F2, and F3, respectively.

*S. aureus* was incubated with untreated control, DA-treated (FD) and cyclotide-modified steel disks (F1–F3), and biofilm observation assayed by crystal violet staining. It is known that biofilm density relates directly to the number of bacteria and is correlated to the staining intensity [26]. Photographic images of the stained biofilms were obtained (Figure 4). Biofilm suspensions were collected after treating stained samples with diluted acetic acid in water. Visually, F1 and F2 showed similar staining intensity to those in untreated control samples, whilst F3 and DA-treated samples showed lower intensity staining.

**Figure 5:**
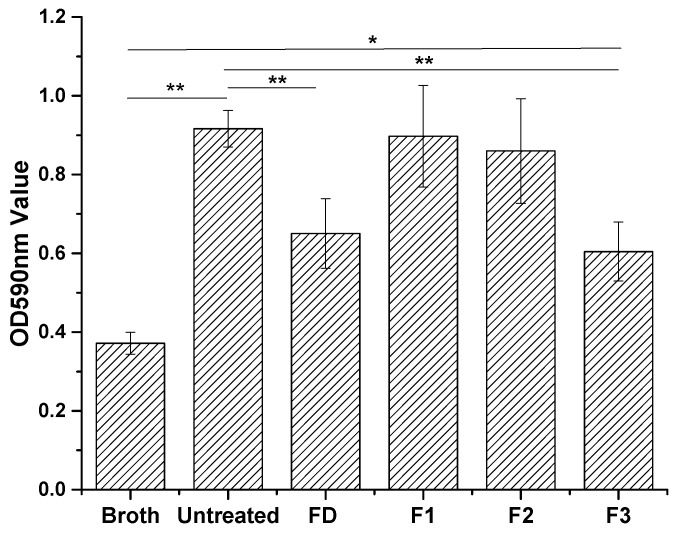
Optical density of the eluted acetic acid from the crystal violet stained metal samples after incubation with *S. aureus* demonstrating the antibiofilm ability of untreated as well as DA- and cyclotide-treated metal surfaces. Metal disk samples treated with DA, P1, P2, and P3 were denoted as FD, F1, F2, and F3, respectively. ANOVA was used to compare groups (* *p* < 0.05; ** *p* < 0.01, *n =* 4). Error bars indicate the standard deviation of the four independent measurements.

Optical density (OD) values of the acetic acid suspensions are presented in Figure 5. Untreated stainless steel gave the highest OD value, indicating the high *S. aureus* biofilm formation for this material (positive baseline). OD values of FD and F3 suspensions were significantly reduced, suggesting antibiofilm properties. F1 and F2, by contrast, did not show significant reduction of biofilm formation.

**Figure 6:**
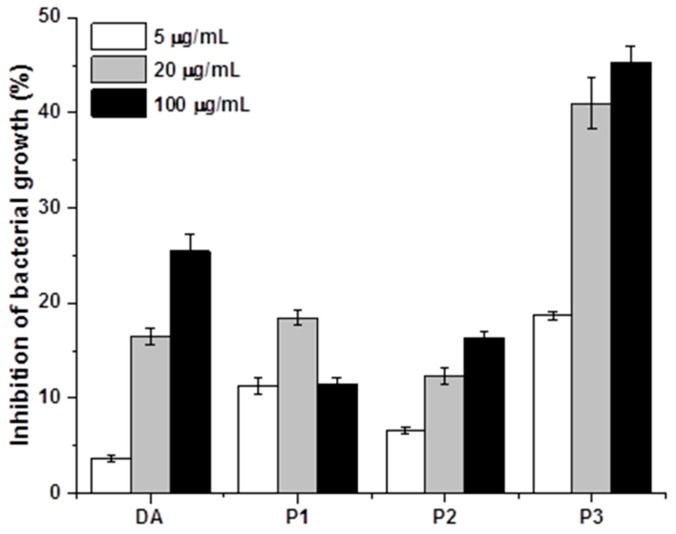
Comparison of the percentage of growth inhibition of *S. aureus* after treatment of DA and cyclotides (P1, a composite of Kalata b1, Varv A, Viba 15, and Viba 17; P2, Varv E; and P3, Viphi G) at three different concentrations (*n* = 3). Error bars indicate the standard deviation of the three independent measurements.

The antibacterial activity of the cyclotides and coupling agent was examined using Alamar blue assay [27]. DA and three cyclotides all demonstrated antibacterial properties (Figure 6). In particular, P3 showed the strongest antibacterial ability compared to DA, P1, and P2. At a concentration of 20 µg/mL of P3, the inhibition of bacterial growth after 24 h incubation with P3 reached more than 40%, while P1, P2, and DA only caused inhibition of 10–20%. However, DA showed slightly better antibacterial ability than P1 and P2 at a higher concentration of 100 µg/mL (Figure 6).

**Figure 7:**
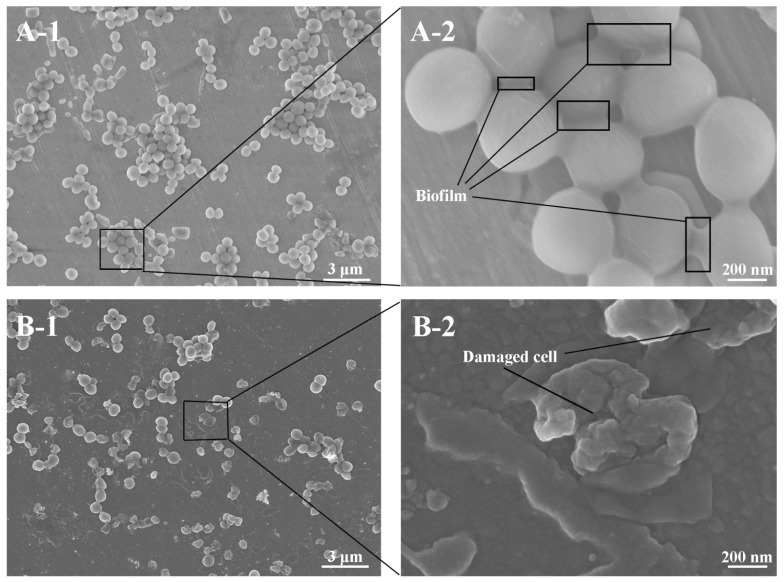
Field emission scanning electron microscopy (FESEM) images of bacteria adhesion on untreated (**A**) and P3 treated samples (**B**).

Field emission scanning electron microscopy (FESEM) was used to directly compare the effects of P3 treatment on biofilm formation (Figure 7). A large number of *S. aureus* were observed to be adhered to untreated samples, with direct observation of biofilm presence (Figure 7(A-2)). F3 surfaces showed fewer adhered bacteria, with multiple damaged bacterial morphologies being seen (Figure 7(B-2)). We attribute these observations to the hydrophobicity and antimicrobial capacity of P3, consistent with the findings from crystal violet staining (Figure 6).

## 3. Discussion

Effective antifouling surface development is of great importance in medicine, food, and transportation. Various methods have been developed to produce a number of antibiofilm surfaces with some advantages and disadvantages [1,2,6,8]. New, low cost, and effective metal surfaces, which do not pose an environmental hazard, remain a pressing need [4,7].

In this study, we explored the possibility of using structurally unique and naturally occurring cyclotides for metal surface modification in order to develop new antifouling materials. To date, hundreds of cyclotides have been isolated from plants, many of which have shown bioactive properties including antimicrobial behavior [22,23,24]. Three major cyclotide fractions (P1, P2, and P3) were isolated from *V. philippica* using HPLC. Mass spectrometry identified these fractions as known cyclotides (Figure 1) with potent cytotoxicity [20]. Solutions of these cyclotides were used to modify stainless steel via a dopamine coupling agent [12,14,15,16,17]. This coupling exploits the presence of nucleophilic side chains of arginine (P1 and P2) and lysine (P3) in cyclotides for a high-efficiency reaction with oxidized polydopamine via Schiff-base reactions [16].

Cyclotide attachment to metal surfaces was confirmed by both FTIR spectroscopy and surface angle analysis. FTIR revealed peaks around 3300, 1690, and 1520 cm^−1^ (Figure 2), corresponding to amide A of N-H stretching; amide Ι of C=O stretching; and amide II of C-N stretching and N-H bending, respectively, which indicated that the peptides bound on the surfaces of stainless steel [28]. Peaks in the region of 1300–1400 cm^−1^ of the FTIR spectra of cyclotide-treated samples were attributed to the presence of disulfide bonds [29]. Contact angle was measured, reflecting the surface wettability of materials. Contact angles of dopamine- and cyclotide-treated samples decreased post-derivatization (Figure 3), again consistent with surface modification by dopamine and cyclotide. The contact angle of the steel sample decreased to 37° ± 1.8° after dopamine modification, similar to the observed value in previous work [30]. The pattern in contact angles of F1–F3 behaved similarly to the predicted hydrophobicity values of the three cyclotides (Table 1). Cyclotide modification of metal surfaces using dopamine as an efficient coupling agent was achieved in a similar manner to previous reports using other peptides/enzymes [15,16,17]. This method was simple, enabling any peptide with available basic residues to attach to metal surfaces. Such simplicity confers significant advantage over other methods of preparing peptide-modified metal surfaces, which require extensive design, protracted chemical synthesis, or expression of specific metal-binding peptide/protein sequences [10,11].

*S. aureus* is a Gram-positive bacterium, widely used as a model for biofilm formation on stainless steel surfaces [15,16]. We employed this model system to study the anti-biofilm properties of cyclotide-modified surfaces. Bacterial presence on stainless steel surfaces was observed visually after staining with crystal violet dye (Figure 4) and measured indirectly by determining OD of acetic acid eluents from the surfaces (Figure 5) [26]. All cyclotides tested induced surface resistance to *S. aureus* biofilm formation, with some variation for different cyclotides. F3 gave comparable antibacterial performance to dopamine, and much higher antibacterial capacity than F1 and F2. Previous studies found a link between bacterial attachment to metal surfaces and damage to *S. aureus* cell membranes, consistent with our results [31]. Contact angle is amongst the factors affecting bacterial attachment. The change of antibiofilm capacity on surfaces treated by different peptides (Figure 4 and Figure 5) is therefore consistent with their contact angle results (Figure 3).

In addition to the impact of cyclotides on bacterial adhesion, we anticipated that the antibacterial properties of cyclotides would further influence bacterial adherence to metal surfaces. The antibacterial properties of adding P1, P2, P3, and DA to bacterial suspensions were therefore assessed (Figure 6). All four compounds inhibited bacterial growth to varying degrees, with dopamine and P3 exerting the strongest inhibitory influence. Therefore, the increased antimicrobial capacity of F3 may be attributed to both surface properties and antimicrobial behavior [23]. FESEM was used to directly confirm the impact of cyclotide P3 on biofilm formation. The morphology of bacteria on the surfaces of untreated samples was compared to F3 (Figure 7). In both cases, clusters of bacteria were observed. The cells of *S. aureus* adhered to untreated stainless steel and were intact and connected by secreted biofilms. On the surfaces treated with the bactericidal cyclotide (F3), many adherent bacteria were lysed, with distorted “wrinkled” morphology. This data supports our hypothesis that cyclotide-modified surfaces possess bactericidal capacity [5].

We investigated the antibiofilm properties of three cyclotide-containing fractions for the modification of metal surfaces. Antibiofilm properties were demonstrated, although the variability suggests that their efficacy could be further optimized. The number of cyclotides with six backbone loops between the conserved cysteine residues may exceed 50,000 in all plant species [32]. The success of this study suggests a ready route for cyclotide isolation and assay from plant-derived materials. Other cyclotides such as cycloviolacin O_2_, whose potent bactericidal activity has been previously demonstrated against Gram-negative bacteria [22], may prove to be good candidates for surface modification. In addition to the use of Gram-positive *S. aureus*, other biofilm-forming Gram-negative bacteria, such as *P. aeruginosa* [22] and marine microorganisms, should also be investigated to broaden the application of cyclotide-modified surfaces.

## 4. Materials and Methods

### 4.1. Materials and Reagents

A total of 304 annealed stainless steel sheets with a thickness of 250 µm were purchased from Wuhan steel factory (Qingshan, China). Stainless steel plates were cut into pieces of Ø10 mm. Stainless steel was cleaned with absolute ethanol for 15 min, immersed into acetone for 1 h, and then air-dried. Crystal violet (CV, 0.1%), methanol, ethyl acetate, dichloromethane, chloroform, dopamine, trifluoroacetic acid (TFA), acetonitrile, Alamar blue, and absolute acetic acid were purchased from Sigma Aldrich (Poole, Dorset, UK). Trypsin-ultra, endo-proteinase GluC, and chymotrypsin (mass spectrometry grade) were purchased from Bio Labs, UK. Formic acid, zip tip (Millipore, Darmstadt, Germany), ammonium bicarbonate (NH_4_HCO_3_), and Tris (2-caboxyethyyl) phosphine hydrochloride (TCEP) were from Thermo Scientific, Loughborough, UK. All reagents were analytical grade. Deionized water was obtained using a Milli-Q water purification system (Merck, Watford, Herts, UK). The aerial parts of *V. philippica* Cav. were collected from Yongchuan, Chongqing, China. The plant was identified by Dr. Qian Wang, Chengdu Institute of Biology, Chinese Academy of Sciences, and a voucher specimen was deposited in the herbarium of Chengdu Institute of Biology, Chinese Academy of Sciences. *S. aureus* ATCC 12,600 was purchased from ATCC (Manassas, VA, USA).

### 4.2. Extraction and Isolation of Cyclotides

The extraction of *V. philippica* crude powder was done using a previously established method with little modification [33]. Briefly, *V. philippica* crude powder (800 g) was pre-extracted with 3.5 L of dichloromethane (DCM) and filtered after 24 h. This was repeated thrice within 96 h. After evaporation of DCM, the DCM extract of *V. philippica* was obtained. The plant residues were dried and macerated with 3.5 L methanol/water (MeOH/H_2_O, 3:2) daily for 96 h. The MeOH/H_2_O crude extract was concentrated using rotary evaporation and subsequently subjected to liquid–liquid partition using n-butanol and H_2_O. Cyclotides were isolated from the butanol fraction using gel column chromatography on a 3 cm i.d. × 42 cm long column loaded with silica gel of 70–230 mesh (Sigma-Aldrich, St. Louis, MO, USA), washing with a mixture of CHCl_3_ and MeOH, increasing the proportion of MeOH until 100% with a rate of 70 drops/min. Cyclotides in the eluted fractions were further purified using semi-preparative HPLC (Agilent 1220 LC, Agilent Technologies, Santa Clara, CA, USA). Separation was achieved using a linear gradient from 10–70% B over 25 mins, where A was 0.1% TFA in water and B 80% acetonitrile containing 0.1% TFA, on a semi-preparative HPLC column (Phenomenex, UK; Jupiter C18 reverse phase, 300 A, 5 μm particle size, 9.4 × 250 mm) at a flow rate of 4 mL/min. Eluted fractions were collected and lyophilized to give purified cyclotides. Three main fractions from *V. philippica* eluted at 18, 19, and 21 min were thus collected and lyophilized, denoted as P1, P2, and P3, respectively.

### 4.3. Mass Spectrometric Analysis of Cyclotides from V. Philippica

#### 4.3.1. Methods for Cyclotides Reduction and Enzyme Digestion

Reduction of cyclotides by TCEP: about 6 nmol of cyclotide (~20 µg) in 20 µL of 0.1 M NH_4_HCO_3_ (pH 8.0) was added to 1 µL of 0.1 M TCEP in the same buffer, and the solution was incubated at 65 °C for 10 min in a similar means as reported [33].

Enzyme digestion: 0.5 µL of trypsin (0.5 µg/µL) was added to the 10 µL reduced peptide (10 µg) to give a final peptide-to-enzyme ratio of 40:1. The trypsin incubation proceeded for 1 h at 37 °C. The digestions were quenched by the addition of an equal volume of 10 µL of 0.5% HCO_2_H and desalted using Zip tips (Millipore). Samples were stored at 4 °C prior to analysis. Chymotrypsin digestion and a combination of trypsin and endoGlu-C digestion were performed in a similar manner as described above.

#### 4.3.2. Liquid Chromatography Electrospray Ionization/Mass Spectrometry (LC ESI/MS)

The cyclotides in both digested and reduced forms were injected onto a Dionex—Ultimate 3000 HPLC system (Thermo Scientific) using a 100% buffer A (5% acetonitrile (MeCN), 0.1% formic acid) in a 20 µL loop. Samples were then separated using a silica C18 column (Acclaim PepMap 100 C18, Thermo Scientific) following a gradient of 0 to 100% buffer B (95% MeCN, 0.1% formic acid) in 60 min. Survey spectra were acquired over the mass range of 400–1600 amu using a Q-ToF Premier mass spectrometer (Waters Corporation, Wilmslow, Cheshire, UK), with automated product ion analysis of multiply-charged precursors with intensity >50 counts per scan. Mass spectra were acquired and analyzed to determine the peptide sequence based on manual de novo sequencing, assisted by the use of PEAKS (Bioinformatics Solutions Inc, Waterloo Ontario, CA, USA) to ascribe b- and y-series ion information using fragments from MS/MS data.

### 4.4. Immobilization of Cyclotides onto the Metal Surfaces

Cyclotides were prepared at 4 mg/mL by dissolution into dimethyl sulfoxide (DMSO), then diluted to 20 µg/mL using 10 mM Tris-HCl buffer (pH 8.5). The polished steel samples were immersed in 20 µg/mL fresh dopamine solution in 10 mM Tris-HCl buffer (pH 8.5) for 12 h. The resulting dopamine-treated metal surfaces (FD) was then covered by cyclotide solutions in a 24-well plate, and incubated with shaking (60 rpm) at room temperature for 24 h. Samples were then removed and rinsed with a large excess of deionized water. All treated samples were air-dried and stored at room temperature for characterization and antibiofilm assay.

### 4.5. Surface Characterization

Contact angle measurements were carried out by the Biolin contact angle instrument using OneAttension software with deionized water. The contact angle was measured within 15 s of placing a 1 µL drop on the surfaces, with an average of five measurements being reported.

Fourier transform infrared spectroscopy (Nicolet AVATAR360) was used to determine the chemical composition of the sample surfaces. FTIR spectra were acquired from 4000 to 800 cm^−1^ with resolution of 4.0 cm^−1^ using the infrared spectrometer equipped with attenuated total reflection (ATR) accessory. Background spectra were acquired and subtracted before sample acquisition, and samples were pressed against the ATR wafer with appropriate pressure to ensure a high signal-to-noise ratio. The spectra for each sample were collected by scanning 256 times and were processed by baseline correction, smoothing, and normalization via the OMNIC software. In the surface characterization process, four duplicates for each group were used in the test to rule out random effect.

### 4.6. Antibiofilm Assay

The amount of biofilms were quantified using the crystal violet staining method [26]. Four replicates of metal samples per group were placed into 24-well suspension culture plates (one sample per well). *S. aureus* bacterial suspension, 1 × 10^6^ CFU/mL (1 mL/well), was added into each well. Plates were sealed with Parafilm^®^ and incubated at 37 °C with continuous shaking (at 60 rpm) for 16 h. The metal samples were removed from the plate and washed four times using sterilized water to remove cell medium and planktonic/non-adhered bacteria. Three-hundred microliters of 0.1% (*w*/*v*) solution of crystal violet in water was added and metal samples stained for ten minutes. Residual solution was then removed and samples were washed four times using sterilized water. Plates were air dried in a fume-hood for ~1 h, before adding 200 µL 30% acetic acid to each well, and recording spectrophotometric absorbance at 590 nm.

### 4.7. Antibacterial (Alamar Blue) Assay

The bacterial isolates were adjusted to McFarland standard 0.5 ((1 − 2) × 10^8^ CFU/mL), and diluted by a factor of 1:100 by addition of 0.2 mL bacterial suspension to 19.8 mL sterile broth medium to give a bacterial concentration of 1× 10^6^ CFU/ml. Diluted *S. aureus* bacterial suspensions were added to 96-well plates (180 µ per well). Cyclotide solutions were added into the bacterial suspension to give a final concentration of 5, 20, and 100 µg/mL (*n* = 3). Plates were incubated 24 h at 37 °C, before adding Alamar blue reagent (20 µL). Fluorescence intensity of the reaction mixture after 4 h incubation at room temperature (RT) was measured using GloMax-plus^®^-Multi detection system (Ex at 525 nm and Em at 580–640 nm). The percentage of inhibition of bacterial cell growth (%) was calculated as (FI_0_ − FI_s_)/FI_0_ × 100, where FI_0_ and FI_s_ were the fluorescence intensity of the bacterial sample without treatment and those after treatment of DA and three cyclotides for 24 h at the three different concentrations.

### 4.8. FESEM Analysis

*S. aureus* suspension with the bacterial concentration of 1 × 10^6^ CFU/mL was prepared in sterile phosphate buffered saline (PBS, pH = 7.4). Untreated metal discs and samples treated with best antibacterial property were put into 24-well plates. The bacterial solution was added into 24-wells (2 mL/well) in a shaker at 37 °C at 120 rpm for 24 h. The samples were taken out and washed three times with sterile PBS to remove the unattached bacteria. The samples were then immersed into 2.5% glutaraldehyde in PBS at 4 °C for 2 h for fixing bacteria. Dehydration of the samples was carried out using 25% (5 min), 50% (5 min), 75% (5 min), 90% (5 min), and 100% (10 min) ethanol solution for FESEM observation (Zeiss Ultra Plus, Germany). The samples were dried in a vacuum at 37 °C before observation. Three duplicates were used for each group.

### 4.9. Statistical Methods

All quantitative measurements were expressed as a mean value ± standard deviation (SD). One-way analysis of variance (ANOVA) was used to compare groups in Excel.

## 5. Conclusions

In summary, cyclotides were isolated and identified from *V. philippica*. These peptides were successfully utilized to modify the surfaces of stainless steel via a simple, versatile, and facile coupling method with dopamine for the first time. It was demonstrated that P3 (Viphi G) was the most effective cyclotide in the antibacterial and antibiofilm aspects after binding to the metal surfaces, compared with P1 and P2. We therefore propose that plant-derived cyclotides, with their unique three-dimensional structure and remarkable stability, can serve as a novel source of biological materials to be used for the modification of metal surfaces for applications in medical devices, aquaculture, food manufacture, and shipbuilding.

## Figures and Tables

**Table 1:** Cyclotides isolated and identified from *V. philippica.*

Peak	Cyclotide	Sequence of Amino Acid Residues	Theoretical Monoisotopic Mass	Experimental Monoisotopic Mass	GRAVY for Their Linear Forms	Net Charges
P1	Varv A Kalata b1 Viba 15 Viba 17	Cyclo-(**C**GET**C**VGGT**C**NTPG---**C**S**C**SWPV**C**TRNGLPV) Cyclo-(**C**GET**C**VGGT**C**NTPG---**C**T**C**SWPV**C**TRNGLPV) Cyclo-(**C**GET**C**VGGT**C**NTPG---**C**A**C**SWPV**C**TRNGLPV) Cyclo-(**C**GET**C**VGGT**C**NTPG---**C**G**C**SWPV**C**TRNGLPV)	2876.17 2890.14 2860.18 2846.02	2876.06 2890.11 2860.12 2846.08	0.148 0.152 0.238 0.162	0 0 0 0
P2	Varv E	Cyclo-(**C**GET**C**VGGT**C**NTPG---**C**S**C**SWPV**C**TRNGLPI)	2890.14	2890.00	0.159	0
P3	Viphi G	Cyclo-(**C**GES**C**VF I P **C** I SAIIG**C**S**C**SNKV**C**YKNGSIP)	3170.43	3170.43	0.726	+1

Note: Cysteine residues (highlighted in bold) form three intramolecular disulfide bonds. The GRAVY (grand average of hydropathicity) values for the corresponding linear forms of each cyclotide were calculated as the sum of hydropathicity values of all the amino acids, divided by the number of residues in the sequence [25]. Higher GRAVY values denote increased hydrophobicity.

## Abbreviations

ANOVA	Analysis of variance
ATR	Attenuated total reflection
CV	Crystal violet
DA	Dopamine
FT-IR	Fourier transform infrared
HPLC	High performance liquid chromatography
GRAVY	Grand average of hydropathicity
OD	Optical density
FESEM	Field emission Electron scanning microscopy
TCEP	Tris (2-caboxyethyyl) phosphine hydrochloride
TFA	Trifluoroacetic acid
